# Supplementary material for: CAP2 is a regulator of actin pointed end dynamics and myofibrillogenesis in cardiac muscle
Source: Commun Biol. 2021 Mar 19;4:365. doi: 10.1038/s42003-021-01893-w (PMC7979805; doi:10.1038/s42003-021-01893-w)
Supplement: Supplementary file 2 — Supplementary Information [file 42003_2021_1893_MOESM2_ESM.pdf]

Supplementary Information

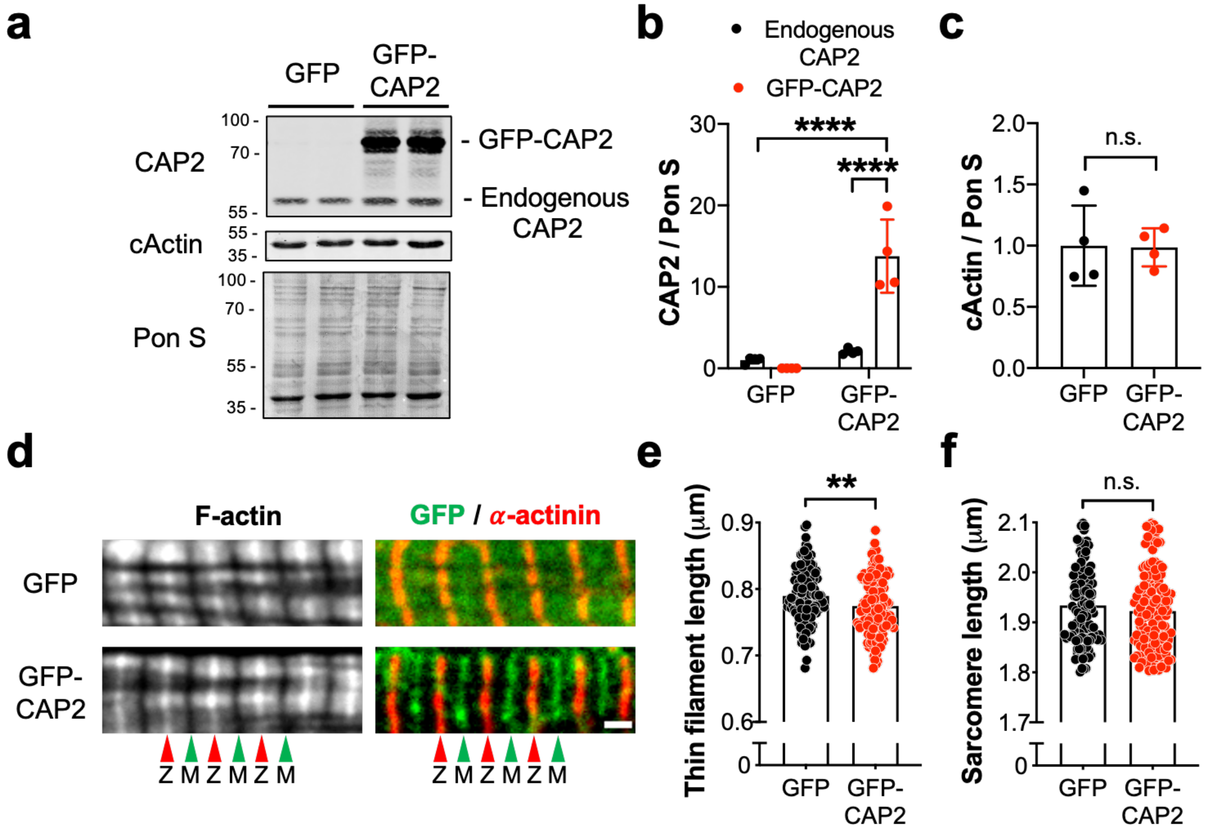

Figure S1. Excess levels of CAP2 have a minor effect on thin filament lengths. a: Immunoblot of lysate from neonatal rat cardiomyocytes transduced with adenovirus expressing GFP or GFP-CAP2 two days after plating and collected four days after plating. **b:** Quantification of immunoblots demonstrate ~15-fold excess of GFP-CAP2 (red dots) over endogenous levels of CAP2 (black dots) (Mean ± SD, n=4 cultures, ****$p<0.0001$, F=31.61, Two-way ANOVA, df=1). **c:** Relative levels of cardiac actin (cActin) in GFP (black dots) or GFP-CAP2-expressing cells (red dots), normalized to total protein by Ponceau S (Pon S) staining (Mean ± SD, n=4 cultures, n.s.: $p=0.9426$, t=0.07506, Student's t-test, df=6). **d:** Immunostaining of GFP or GFP-CAP2-expressing cells was performed using anti-α-actinin (red) antibody and phalloidin to probe for F-actin. The locations of Z-discs (Z) and pointed ends near the M-line (M) are indicated. Scale bar = 1 μm. **e:** Thin filament lengths and **f:** sarcomere lengths measured from cells transduced adenovirus expressing GFP (black dots) or GFP-CAP2 (red dots) within a sarcomere length range of 1.8-2.1 μm. Thin filaments of GFP-CAP2-expressing cells were only ~2% shorter than that of GFP-expressing cells (Mean ± SEM, n=128, 157 total measurements from 10 cells per culture, 3 cultures; **$p=0.0025$, t=3.052; n.s.: $p=0.2156$, t=1.241, Student's t-test, df=283, n.s.: not significant).

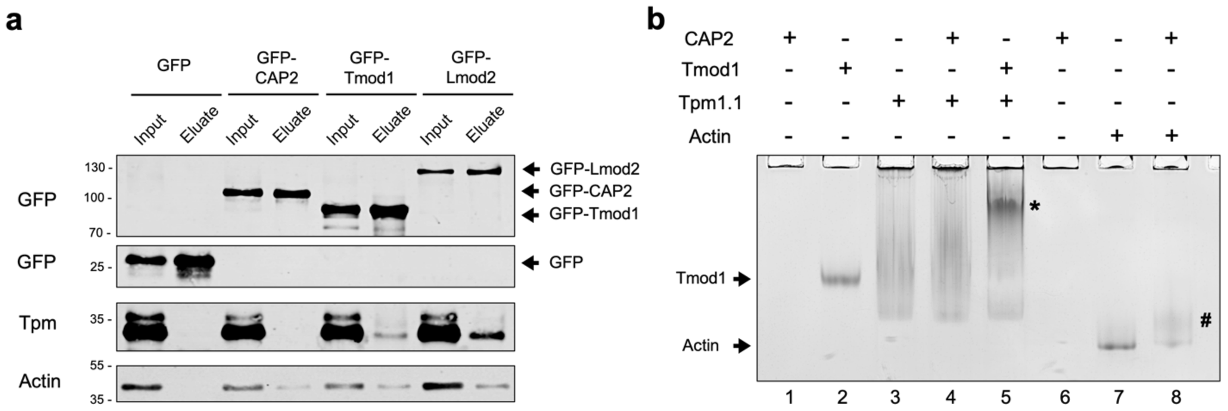

Figure S2. CAP2 does not interact with Tpm. a: Neonatal rat cardiomyocytes were transduced with adenovirus expressing GFP, GFP-CAP2, GFP-Tmod1 or GFP-Lmod2 two days after plating and lysed four days after plating for pull-down of GFP-tagged proteins using GFP-affinity beads. Immunoblot analysis demonstrates pull-down of Tpm with GFP-Tmod1 and GFP-Lmod2 as expected, but no pull-down with GFP-CAP2. Actin was pulled-down by all proteins except GFP-only (control). **b:** 9% acrylamide gels in the absence of SDS were used to assess the binding between 3 μM CAP2 or Tmod1 and 6 μM Tpm1.1 or 3 μM actin. A complex between Tmod1 and Tpm1.1 (*, lane 5), and actin and CAP2 (#, lane 8) was observed to form. CAP2 and Tpm1.1 were not observed to interact (lane 4). Note, CAP2 (lanes 1 and 6) did not enter the gel likely due to its oligomeric nature and/or isoelectric point.

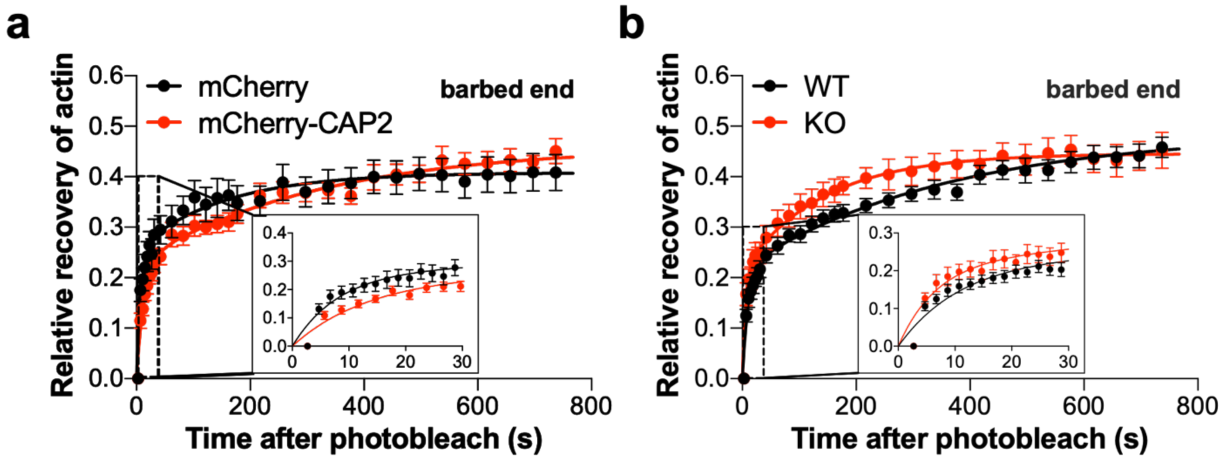

Figure S3. The effect of CAP2 levels on actin incorporation at thin filament barbed ends. Mean relative recovery of GFP-cardiac actin at thin filament barbed ends of **a:** mCherry or mCherry-CAP2-expressing neonatal rat cardiomyocytes, and **b:** neonatal WT or *Cap2*-KO mouse cardiomyocytes after photobleaching over time (Mean ± SEM, n=11, 13, 11, 11 cells from three independent cultures).

Table S1. Summary of FRAP data from mCherry or mCherry-CAP2-expressing cells

	Barbed end			Pointed end			
	**mCherry**	**mCherry-CAP2**	***p* value**	**mCherry**	**mCherry-CAP2**	***p* value**	***F* value**
n	11	7	-	13	10	-	-
M_{fast}	0.31 ± 0.06	0.23 ± 0.02	0.8073	0.52 ± 0.11	**0.22 ± 0.02***	0.0106	8.769
k_{fast} (s^{-1})	0.143 ± 0.035	0.099 ± 0.024	0.9671	0.441 ± 0.092	**0.078 ± 0.011*****	0.0006	10.28
$t_{1/2fast}$ (s)	7.0 ± 1.7	7.8 ± 2.0	0.9861	1.6 ± 0.3	**9.8 ± 1.4****	0.0012	9.334
M_{slow}	0.20 ± 0.02	0.25 ± 0.03	0.6201	0.23 ± 0.02	0.34 ± 0.05	0.0641	7.065
k_{slow} (s^{-1})	0.005 ± 0.001	0.003 ± 0.001	0.8909	0.008 ± 0.001	**0.004 ± 0.001***	0.0204	6.357
$t_{1/2slow}$ (s)	132.5 ± 26.8	194.4 ± 35.8	0.4548	104.5 ± 16.7	201.3 ± 41.6	0.1086	7.261
M_{fast} + M_{slow}	0.50 ± 0.07	0.49 ± 0.04	0.9999	0.75 ± 0.101	0.58 ± 0.06	0.4016	1.351

Recovery data were best fit using nonlinear regression curves with a two-exponential association equation {$R = M_{fast} \times [1 - \exp(-k_{fast} \times t)] + M_{slow} \times [1 - \exp(-k_{slow} \times t)]$}. R is the relative recovery of fluorescence at time t. Mean mobile fraction (M), half-time of recovery ($t_{1/2}$) and rate constants (k) are indicated for both slow and fast components of recovery ± SEM (n=7-13 cells from three independent cultures). The total mobile fraction is the sum of M_{fast} and M_{slow}. Bolded text indicates values that are statistically significantly different between mCherry and mCherry-CAP2 (Two-way ANOVA, df=1).

Table S2. Summary of FRAP data from WT and *Cap2*-KO cells

	**Barbed end**			**Pointed end**			
	**WT**	**KO**	***p* value**	**WT**	**KO**	***p* value**	***F* value**
n	11	10	-	11	8	-	-
M_{fast}	0.24 ± 0.03	0.21 ± 0.02	0.6381	0.27 ± 0.03	0.25 ± 0.02	0.8832	0.8319
k_{fast} (s^{-1})	0.101 ± 0.010	**0.153 ± 0.010****	0.0012	0.106 ± 0.010	**0.142 ± 0.011***	0.0356	19.10
$t_{1/2fast}$ (s)	7.7 ± 0.9	**4.7 ± 0.4***	0.0229	7.5 ± 1.2	5.2 ± 0.4	0.0993	10.85
M_{slow}	0.29 ± 0.02	0.23 ± 0.02	0.1089	0.35 ± 0.01	0.29 ± 0.02	0.0923	8.119
k_{slow} (s^{-1})	0.003 ± 0.001	**0.007 ± 0.001******	<0.0001	0.003 ± 0.001	**0.007 ± 0.001****	0.0012	40.31
$t_{1/2slow}$ (s)	264.1 ± 28.9	**105.2 ± 10.6*****	0.0004	261.1 ± 37.8	**125.2 ± 19.5****	0.0042	27.41
M_{fast} + M_{slow}	0.53 ± 0.04	0.45 ± 0.03	0.1914	0.61 ± 0.03	0.55 ± 0.03	0.3352	4.590

Recovery data were best fit using nonlinear regression curves with a two-exponential association equation $\{R = M_{fast} \times [1 - \exp(-k_{fast} \times t)] + M_{slow} \times [1 - \exp(-k_{slow} \times t)]\}$. R is the relative recovery of fluorescence at time t. Mean mobile fraction (M), half-time of recovery ($t_{1/2}$) and rate constants (k) are indicated for both slow and fast components of recovery ± SEM (n=9-11 cells from three independent cultures). The total mobile fraction is the sum of M_{fast} and M_{slow}. Bolded text indicates values that are statistically significantly different between WT and KO cells (Two-way ANOVA, df=1).

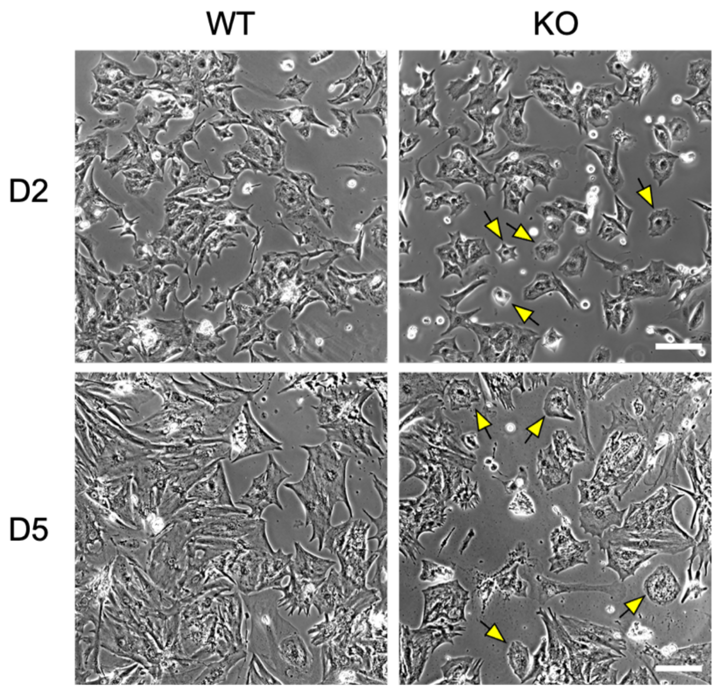

Figure S4. *Cap2*-KO cells appear rounder and less developed than WT cells. Cardiomyocytes were cultured from the hearts of neonatal WT or *Cap2*-KO mice and imaged live using a bright field microscope 2 or 5 days after plating (D2, D5). Yellow arrows indicate *Cap2*-KO cells that appear rounder and less rod-shaped compared to WT cells. Scale bar = 100 µm.

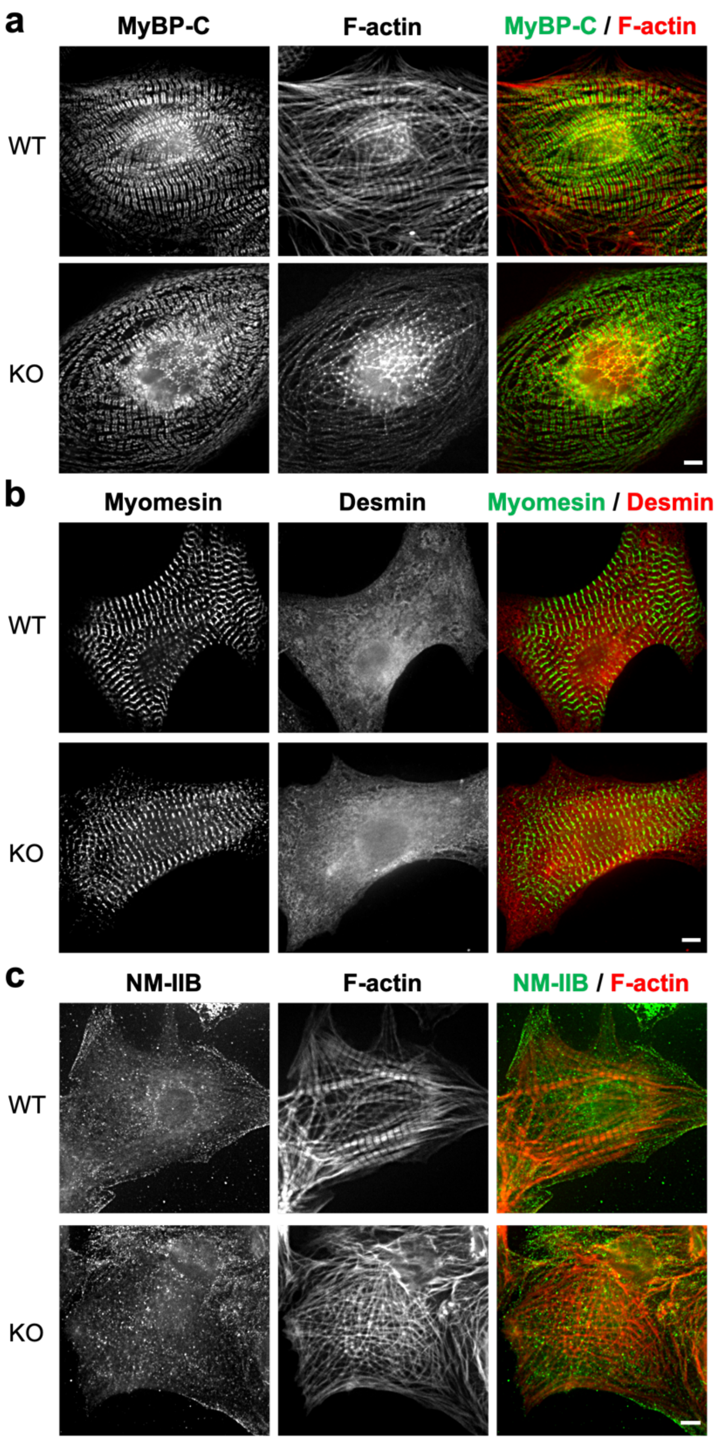

Figure S5. *Cap2*-KO cells have normal assembly of thick filament proteins and other sarcomeric markers of cardiomyocyte maturation. Cardiomyocytes were cultured from the hearts of neonatal WT or *Cap2*-KO mice and fixed 1 day after plating for immunofluorescence analysis. Staining of WT or KO cells was done using **a:** anti-myosin-binding protein C (MyBP-C, green) antibody and phalloidin (red) or **b:** anti-myomesin (green) and anti-desmin (red) antibodies, or **c:** anti-nonmuscle myosin IIB (NM-IIB, green) antibody and phalloidin (red) to probe for F-actin**.** Scale bar = 5 μm.

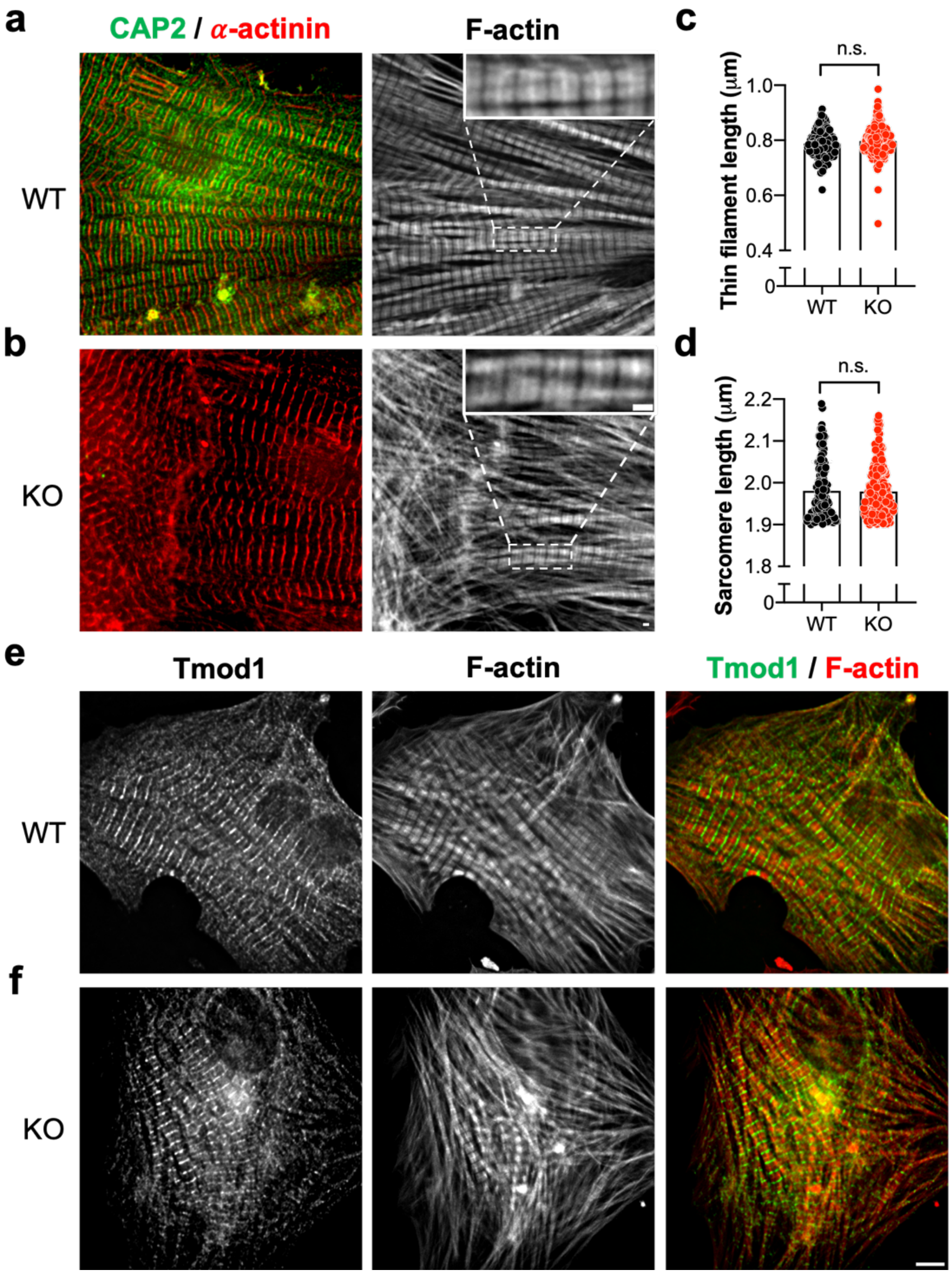

Figure S6. *Cap2*-KO cardiomyocytes have comparable thin filament lengths with WT cardiomyocytes. Cardiomyocytes derived from neonatal WT or *Cap2*-KO mice were fixed 5 days after plating. Immunostaining of **a:** WT and **b:** *Cap2*-KO cardiomyocytes was done using anti-CAP2 (green) and anti-α-actinin (red) antibodies, and phalloidin to probe for F-actin. Scale bar = 1 µm. **c:** Thin filament lengths and **d:** sarcomere lengths of WT (black dots) or *Cap2*-KO cells (red dots) measured within a sarcomere length range of 1.9-2.2 µm (Mean ± SEM, n=136, 296 total measurements from 10 cells per culture, 3 cultures, n.s.: p=0.0850, t=1.726; n.s.: p=0.7926, t=0.2630, Student's t-test, df=430, n.s.: not significant). Neonatal **e:** WT or **f:** *Cap2*-KO mouse cardiomyocytes were stained using anti-Tmod1 (green) and phalloidin (red) to probe for F-actin. Tmod1 assembly at the pointed ends was similar between WT and *Cap2*-KO cells. Scale bar = 5 µm.

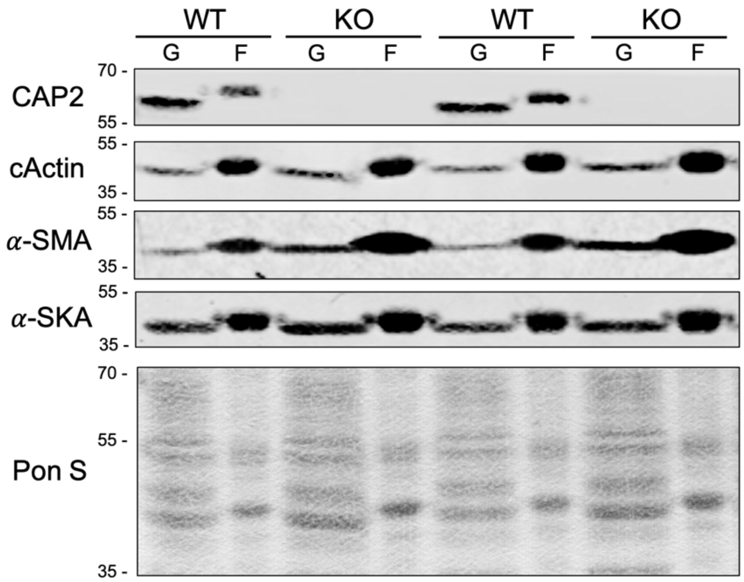

Figure S7. Immunoblots for the globular (G) and filamentous (F) populations of cardiac actin (cActin), α-smooth muscle actin (α-SMA) and α-skeletal muscle (α-SKA) in WT or *Cap2*-KO cells. Immunoblots show unchanged levels of globular and filamentous cActin but increased levels of globular and filamentous α-SMA and α-SKA in *Cap2*-KO cells. Note, the percentage of actin was not quantified since the total expression level of α-SMA and α-SKA is increased in *Cap2*-KO cells.

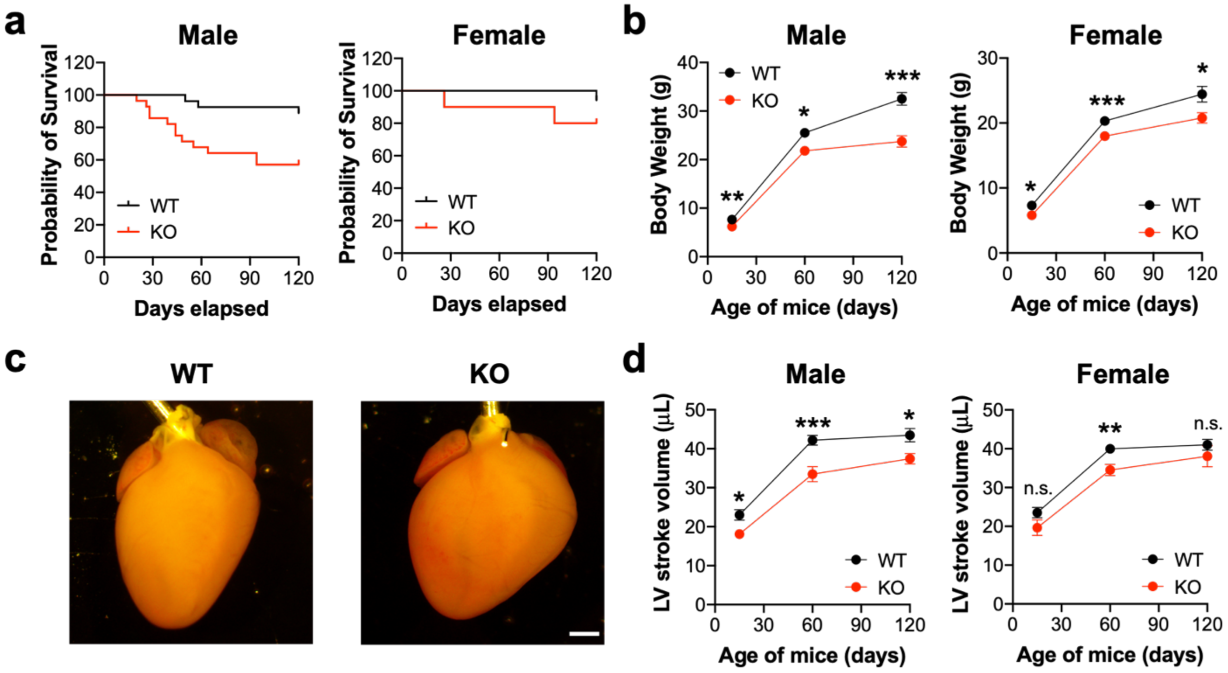

Figure S8. *Cap2*-KO mice present with dilated cardiomyopathy and sudden cardiac death. a: Survival curve of WT (black line) or *Cap2*-KO (red line) male and female mice. The curve of male (p=0.0062, χ^2=7.492, df=1), but not female (p=0.2417, χ^2=1.371, df=1) *Cap2*-KO mice is significantly different from WT mice, with male mice having a significantly shorter lifespan (n=27, 28, 17, 18 mice, log-rank test). **b:** Body weight is reduced in male (n=10 mice, **p=0.0027, t=3.468, df=18; n=8, 9 mice, *p=0.0134, t=2.802, df=15; n=8, 10 mice ***p=0.0001, t=5.015, df=16) and female (n=10 mice, *p=0.0209, t=2.530, df=18; n=10, 11 mice ***p=0.0005, t=4.207, df=19; n=11, 9 mice *p=0.0350, t=2.291, df=17) *Cap2*-KO mice (red) compared to WT mice (black) (Mean ± SEM, Student's t-test). **c:** Representative images of WT or *Cap2*-KO male hearts on postnatal day 15. Scale bar = 1 mm. **d:** Echocardiography analysis reveals a consistent reduction in the left ventricular (LV) stroke volume in the hearts of *Cap2*-KO (red) male (n=21, 11 mice, *p=0.0175, t=2.515, df=30; n=28, 14 mice ***p=0.0004, t=3.874, df=40; n=14, 9 mice *p=0.0203, t=2.511, df=21), but not in the female mice (n=15, 12 mice, n.s.: p=0.1112, t=1.654, df=24; n=29, 14 mice **p=0.0019, t=3.328, df=41; n=22, 7 mice *p=0.3229, t=1.007, df=27) compared to WT mice (black) (Mean ± SEM, Student's t-test, n.s.: not significant).

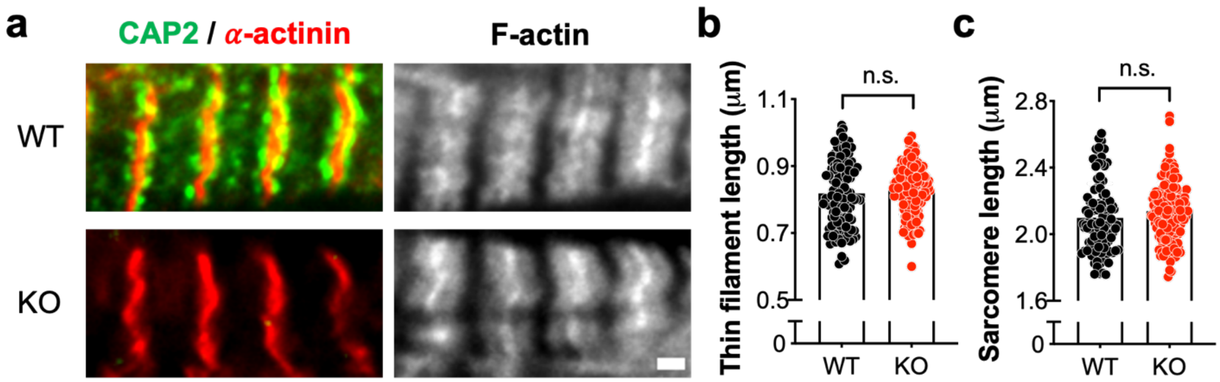

Figure S9. Thin filament lengths are comparable in WT and *Cap2*-KO mice. Left ventricular tissue isolated from WT or *Cap2*-KO mice were stretched and fixed for immunofluorescence analysis. **a:** Immunostaining of WT or *Cap2*-KO hearts was done using anti-CAP2 (green) and anti-α-actinin (red) antibodies, and phalloidin to probe for F-actin. Scale bar = 1 µm. **b:** Thin filament lengths and **c:** sarcomere lengths between the WT (black dots) and *Cap2*-KO hearts (red dots) were not statistically significantly different (Mean ± SEM, n=104, 170 total measurements from 10 cells per mouse, 3 mice, n.s.: p=0.3574, t=0.9219; n.s.: p=0.0744, t=1.791, Student's t-test, df=272, n.s.: not significant).

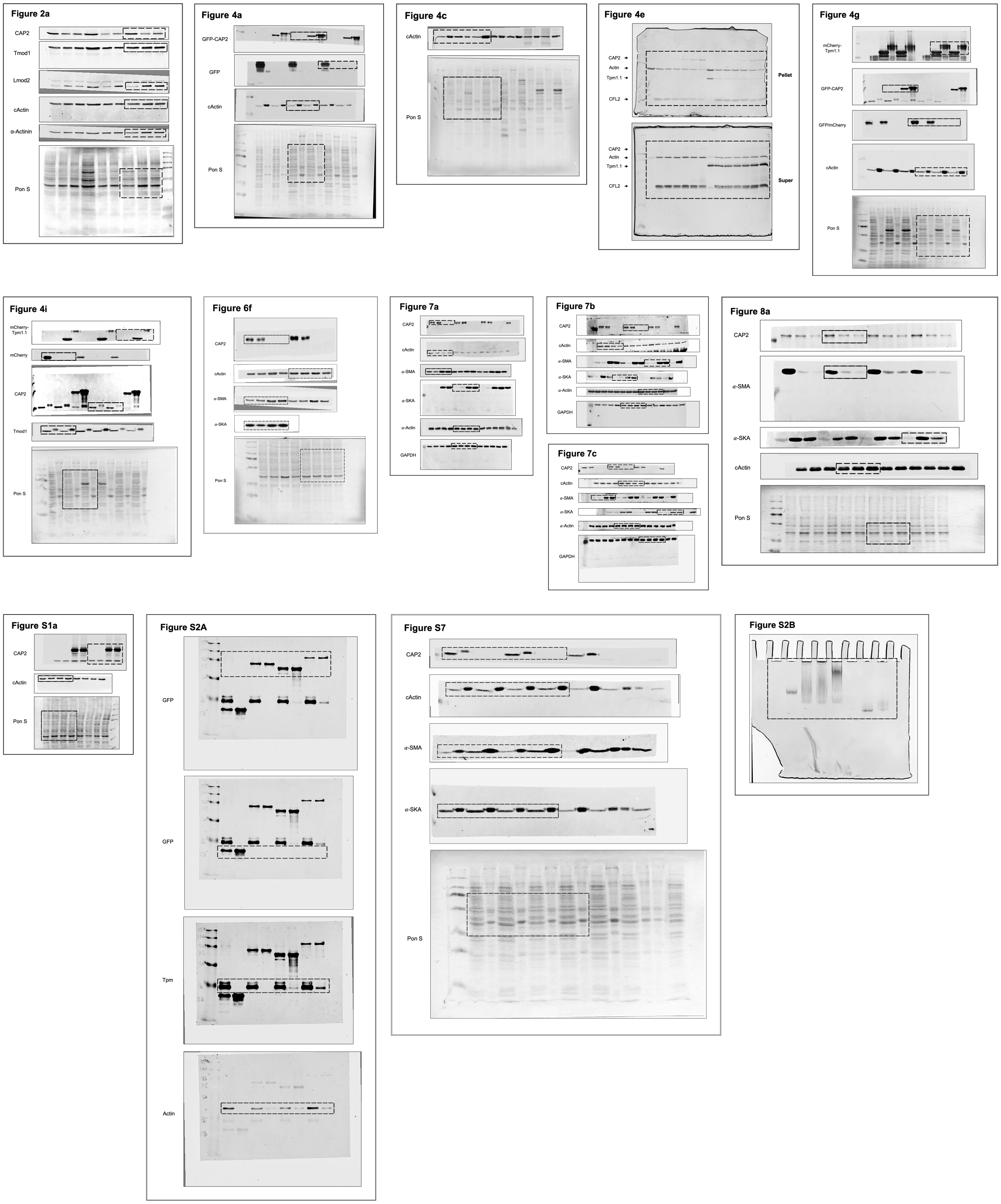

Figure S10. Uncropped gels and blots for all figures. The areas used in the figures are marked by dashed boxes.
