# Supplementary material for: CAP2 is a regulator of actin pointed end dynamics and myofibrillogenesis in cardiac muscle
Source: Commun Biol. 2021 Mar 19;4:365. doi: 10.1038/s42003-021-01893-w (PMC7979805; doi:10.1038/s42003-021-01893-w)
Supplement: Supplementary file 3 — Description of Additional Supplementary Files [file 42003_2021_1893_MOESM3_ESM.pdf]

## Description of Additional Supplementary Files

**File name:** Supplementary Data 1

**Description:** Source data for Figures 1-8.
