# Supplementary material for: CAP2 is a regulator of actin pointed end dynamics and myofibrillogenesis in cardiac muscle
Source: Commun Biol. 2021 Mar 19;4:365. doi: 10.1038/s42003-021-01893-w (PMC7979805; doi:10.1038/s42003-021-01893-w)
Supplement: Supplementary file 5 — Reporting Summary [file 42003_2021_1893_MOESM5_ESM.pdf]

nature research

Corresponding author(s): Carol C. Gregorio

Last updated by author(s): Feb 4, 2021

Reporting Summary

Nature Research wishes to improve the reproducibility of the work that we publish. This form provides structure for consistency and transparency in reporting. For further information on Nature Research policies, see our Editorial Policies and the Editorial Policy Checklist.

Statistics

For all statistical analyses, confirm that the following items are present in the figure legend, table legend, main text, or Methods section.

n/a	Confirmed	
[ ]	[x]	The exact sample size (*n*) for each experimental group/condition, given as a discrete number and unit of measurement
[ ]	[x]	A statement on whether measurements were taken from distinct samples or whether the same sample was measured repeatedly
[ ]	[x]	The statistical test(s) used AND whether they are one- or two-sided<br>*Only common tests should be described solely by name; describe more complex techniques in the Methods section.*
[x]	[ ]	A description of all covariates tested
[ ]	[x]	A description of any assumptions or corrections, such as tests of normality and adjustment for multiple comparisons
[ ]	[x]	A full description of the statistical parameters including central tendency (e.g. means) or other basic estimates (e.g. regression coefficient) AND variation (e.g. standard deviation) or associated estimates of uncertainty (e.g. confidence intervals)
[ ]	[x]	For null hypothesis testing, the test statistic (e.g. *F*, *t*, *r*) with confidence intervals, effect sizes, degrees of freedom and *P* value noted<br>*Give P values as exact values whenever suitable.*
[x]	[ ]	For Bayesian analysis, information on the choice of priors and Markov chain Monte Carlo settings
[x]	[ ]	For hierarchical and complex designs, identification of the appropriate level for tests and full reporting of outcomes
[x]	[ ]	Estimates of effect sizes (e.g. Cohen's *d*, Pearson's *r*), indicating how they were calculated

Our web collection on statistics for biologists contains articles on many of the points above.

Software and code

Policy information about availability of computer code

Data collection	ImageJ Version 1.52 (NIH) was used for collecting data from SDS- or nondenaturing-PAGE gels and images of cardiac actin immunostain. ZEN software 2.3 Blue Edition (Zeiss) was used for acquiring super-resolution microscopy data. Image J Version 1.52 DDecon plug-in was used for thin filament length measurements. Data from immunoblot membranes were collected using Image Studio Lite Version 5.2.5 (LI-COR Biosciences). FRAP data were acquired with Leica LAS X 3.4.2.18368. Echocardiography data were collected with Vevo Lab 3.2.6 (VisualSonics).
Data analysis	GraphPad Prism 8.4.1 was used for compiling the data, creating the figures and statistical analysis. Fluorescence recovery data from FRAP experiments were analyzed with Leica LAS X 3.4.2.18368. Echocardiography data were analyzed with Vevo Lab 3.2.6 (VisualSonics).

For manuscripts utilizing custom algorithms or software that are central to the research but not yet described in published literature, software must be made available to editors and reviewers. We strongly encourage code deposition in a community repository (e.g. GitHub). See the Nature Research guidelines for submitting code & software for further information.

Data

Policy information about availability of data

All manuscripts must include a data availability statement. This statement should provide the following information, where applicable:

- Accession codes, unique identifiers, or web links for publicly available datasets
- A list of figures that have associated raw data
- A description of any restrictions on data availability

The datasets generated during and/or analyzed during the current study are available from the corresponding author on reasonable request.

Field-specific reporting

Please select the one below that is the best fit for your research. If you are not sure, read the appropriate sections before making your selection.

[x] Life sciences [ ] Behavioural & social sciences [ ] Ecological, evolutionary & environmental sciences

For a reference copy of the document with all sections, see nature.com/documents/nr-reporting-summary-flat.pdf

Life sciences study design

All studies must disclose on these points even when the disclosure is negative.

Sample size	For the cell culture and recombinant protein studies, we initially aimed to perform 3 independent experiments in this manuscript. If data from a certain group(s) failed to reach to a sample size of 3 due to an inability of quantification resulting from technical difficulties (imperfections in gel/staining/culturing), the experiment was repeated for a fourth time in order to reach to a sample size of at least 3 for all groups and to achieve statistical significance.<br>In general, most of the physiological studies carried out in our Genetically Engineered Mouse Model core facility have been achieved with 6 animals of any given genotype per experiment. Therefore in order to achieve the statistical significance level of p<0.05, 6-8 mice per genotype per gender were used. Data including more than 8 mice (i.e. echocardiography and body weight data on postnatal days 15 or 60) resulted from taking measurements from the same mice that were grown to postnatal day 120 at different ages. We chose not to discard any collected data, therefore sample size varied in those particular experiments.
Data exclusions	The only data exclusion was done in FRAP and thin filament length measurements. If fitting the two-phase exponential equation to the fluorescence recovery data did not provide an R2 value greater than 0.70 or did not converge, the calculated parameters were deemed unreliable and not included in analysis. Similarly, for thin filament length measurements if the fit equation did not converge, that data set was discarded.
Replication	All attempts of replication was successful. To demonstrate this statement, we exhibit all individual data points in the figures, where applicable.
Randomization	Samples or organisms were allocated into groups based on treatment, genotype, gender and age. For cell culture experiments, control treated (Buffer, DMSO, GFP or mCherry) cells were grouped and compared to the treatment of interest. For animal experiments, wild-type or knockout mice were categorized separately and comparisons were performed only within the same gender and age.
Blinding	Blinding was performed where applicable. For example, if the treatment type of a subject could be distinguished (i.e. genotyping in mice) after data collection and analysis, the groups were blinded. When applicable, folders containing data sets were blinded and revealed after analysis (i.e. FRAP and thin filament length measurements). For certain applications (immunoblots and immunofluorescence) blinding was not possible since the treatment (i.e. presence of GFP fluorescence or the CAP2 gene) had to be determined prior to collecting data.

Reporting for specific materials, systems and methods

We require information from authors about some types of materials, experimental systems and methods used in many studies. Here, indicate whether each material, system or method listed is relevant to your study. If you are not sure if a list item applies to your research, read the appropriate section before selecting a response.

Materials & experimental systems

n/a	Involved in the study
[ ]	[x] Antibodies
[ ]	[x] Eukaryotic cell lines
[x]	[ ] Palaeontology and archaeology
[ ]	[x] Animals and other organisms
[x]	[ ] Human research participants
[x]	[ ] Clinical data
[x]	[ ] Dual use research of concern

Methods

n/a	Involved in the study
[x]	[ ] ChIP-seq
[x]	[ ] Flow cytometry
[x]	[ ] MRI-based neuroimaging

Antibodies

Antibodies used	Rabbit polyclonal anti-CAP2 (15865-1-AP, Proteintech, Rosemont, IL), mouse monoclonal anti-a-actinin (EA53, Sigma-Aldrich, St. Louis, MO), rabbit polyclonal anti-Tmod1 (custom made) , mouse monoclonal anti-myomesin (B4, a kind gift from Dr. Elizabeth Ehler, King's College, London, UK), mouse monoclonal anti-α-smooth-muscle-actin-FITC (clone 1A4, Sigma-Aldrich), mouse monoclonal anti-α-skeletal-muscle-actin (MUB0108P, Exalpha Biologicals Inc.), rabbit polyclonal anti-myosin binding protein-C (Myomedix, Germany), rabbit polyclonal anti-desmin (Biomeda, Foster City, CA) and mouse monoclonal nonmuscle myosin IIB (CMII 25, Developmental Studies Hybridoma Bank), rabbit polyclonal anti-Lmod2 (E13, Santa Cruz Biotechnology, Dallas, Texas), mouse monoclonal anti-cardiac actin (1:1,000) (03-61075, American Research Products, Inc., Waltham, MA), goat polyclonal anti-a-actinin (1:400) (AF8279, R&D Systems, Minneapolis, MN), mouse monoclonal anti-GFP (B-2, Santa Cruz Biotechnology), mouse monoclonal anti α-smooth-muscle-actin (A-2547, Sigma-Aldrich), mouse monoclonal anti-α-muscle-actin (MUB0107P, Exalpha Biologicals Inc.), mouse

	monoclonal anti-tropomyosin-1 (TM311, Novus Biologicals), rabbit polyclonal anti-pan actin (AAN01-A, Cytoskeleton, Inc.) and mouse monoclonal anti-GAPDH (clone 6C5; Life Technologies).
Validation	We have tested a few different antibody products against each protein. The tested antibodies were first picked based on the manufacturers' recommendations on cross-reactivity and specificity. We further validated the antibodies by testing them by both immunostaining or immunoblotting. We observed certain antibodies to produce signal in one application but not in the other, therefore we limited their use to certain applications. Known molecular weights of detected proteins were used as an indicator of specificity in immunoblots. For immunostaining of proteins that have known cellular localizations, we compared our findings to published data to validate our results. Secondary antibodies alone were always used to determine the background signal. Antibodies against CAP2, Lmod2 and Tmod1 did not produce any signal in the tissues of the respective KO mouse models for these proteins by both immunofluorescence and immunoblotting.

Eukaryotic cell lines

Policy information about cell lines

Cell line source(s)	Neonatal rat, mouse or chick ventricular tissue for primary cardiomyocyte cultures.
Authentication	None of the cell lines used were authenticated.
Mycoplasma contamination	Cell lines were not specifically tested against mycoplasma contamination. However, for each culture cells were monitored by light microscopy daily and if contamination was suspected (based on observation of small, circular cells and subsequent disruption of cardiomyocyte beating), the cultures were discarded.
Commonly misidentified lines (See ICLAC register)	No misidentified cell lines were used.

Animals and other organisms

Policy information about studies involving animals; ARRIVE guidelines recommended for reporting animal research

Laboratory animals	Mixed gender Sprague-Dawley rats, C57BL/6J mice and day 6 embryonic chicks (eggs) were used for cardiomyocyte cultures. Both male and female WT or Cap2-KO mice with C57BL/6J background were investigated for body weight and stroke volume measurements. Male mice were used for thin filament length measurements, immunofluorescence and immunoblot analysis since the disease phenotype is more apparent in male mice as reported previously and in our manuscript.
Wild animals	This study did not involve wild animals.
Field-collected samples	The study did not involve samples collected from the field.
Ethics oversight	Work with animals was performed under the approval by The Institutional Animal Care and Use Committee at the University of Arizona; Protocol number 08-017, which conformed to all applicable federal and institutional policies, procedures and regulations, including the PHS Policy on Humane Care and Use of Laboratory Animals, USDA regulations (9 CFR Parts 1, 2, 3), the Federal Animal Welfare Act (7 USC 2131 et. Seq.), the Guide for the Care and Use of Laboratory Animals, and all relevant institutional regulations and policies regarding animal care and use at the University of Arizona.

Note that full information on the approval of the study protocol must also be provided in the manuscript.
